# A multicenter cohort study to investigate the factors associated with functional autonomy change in patients with cognitive complaint or neurocognitive disorders: the MEMORA study protocol

**DOI:** 10.1186/s12877-019-1204-1

**Published:** 2019-07-18

**Authors:** Virginie Dauphinot, Claire Moutet, Isabelle Rouch, Mathieu Verdurand, Christelle Mouchoux, Floriane Delphin-Combe, Sylvain Gaujard, Pierre Krolak-Salmon, the MEMORA group, Pierre Krolak-Salmon, Virginie Dauphinot, Florian Delphin-Combe, Zaza Makaroff, Denis Federico, Marie-Hélène Coste, Isabelle Rouch, Jean-Michel Dorey, Alexis Lepetit, Keren Danaila, Julien Vernaudon, Anthony Bathsavanis, Alain Sarciron, Yves Guilhermet, Sylvain Gaujard, Pierre Gromaître, Claire Moutet, Mathieu Verdurand

**Affiliations:** 1 Centre Mémoire Ressource et Recherche de Lyon (CMRR), Hôpital des Charpennes, Hospices Civils de Lyon, Lyon, France; 2 Institut du Vieillissement I-Vie, Hospices Civils de Lyon, Lyon, France; 3 Centre Mémoire Ressource et Recherche de Saint Etienne (CMRR), service de neurologie, CHU de Saint-Etienne, Saint-Etienne, France; 4 Centre de Recherche Clinique CRC - VCF (Vieillissement – Cerveau - Fragilité), Hôpital des Charpennes, Hospices Civils de Lyon, Lyon, France; 5 INSERM, U1028; CNRS, UMR5292; Lyon Centre de Recherche en Neurosciences de Lyon, Dynamique Cérébrale et Cognition, Lyon, France; 6 Hôpital des Charpennes, 27 rue Gabriel Péri, 69100 Villeurbanne, France

**Keywords:** Neurocognitive disorders, Activity of daily living, Longitudinal study, Memory

## Abstract

**Background:**

The identification of factors associated with functional impairment, in particular those which are potentially modifiable, may help to delay the advanced stages of functional dependence in patients with neurocognitive disorders such as Alzheimer’s disease and related disorders.

The objectives of the MEMORA cohort are to investigate the factors associated, first with functional autonomy change over time, and secondarily with the cognitive performance and behavioral disorders changes over time.

**Methods:**

The MEMORA study is a multicenter prospective cohort study carried out throughout the patient’s care pathway, in Memory centers of Lyon (France). The study will include 6780 patients at all stages of memory disorders in 6 years. The follow-up for each patient is planned for 3 years. The main outcome is the functional autonomy level change as assessed by the instrumental abilities of daily living (IADL) score. Patient characteristics include sociodemographic and clinical features, neuropsychological performance, pharmaceutical and non-pharmaceutical therapy.

**Discussion:**

This study conducted in a context of routine care may help to identify the factors associated with functional impairment related to progressive neurocognitive disorders. Subsequently, interventions on potentially modifiable factors could be proposed to the patients to improve their management and delay functional dependence.

**Trial registration:**

NCT02302482, registered 27 November 2014.

## Background

Alzheimer’s disease and related disorders (ADRD) result in cognitive loss that progress towards functional loss of autonomy, and behavioral disorders [1, 2]. The decline of functional abilities and cognitive performance, as well as a behavioral disturbance are among the main predictors of nursing home placement, and therefore represent an important burden for the family and the society, and a public health stake [3]. Previous studies have identified a number of potential risk factors for ADRD, major neurocognitive disorders (NCD), or cognitive decline i.e. high blood pressure, vascular risk factors, stroke, arterial fibrillation, diabetes mellitus, hypercholesterolemia, hyperhomocysteinemia, biological inflammation, genetic biomarkers, obesity, sedentary lifestyle, smoking habit, depression, educational level [1, 4–6]. Nevertheless, the role of some of these factors on the progression of the NCD remains unclear [7, 8]. In addition, there is little published data on to the risk factors for loss of functional autonomy in patients at all stages of NCD. Indeed, the previous published studies have considered either community-dwelling elderly subjects without NCD at inclusion, or patients with AD among whose functional autonomy was already altered [9–12].

As research for a curative treatment of ADRD continues, identifying and improving the understanding of the factors implicated in the progression of functional disability could allow to develop and propose interventions to target potentially modifiable factors with the perspective to prevent or slow the functional disability in patients with NCD [13, 14].

The MEMORA cohort, including patients followed in memory centers (MC), has been designed to study the determinants for functional decline of patients at all stage of memory troubles including subjective cognitive decline (SCD), and minor or major neurocognitive disorders (NCD) [15, 16].

In this manuscript, the MEMORA study protocol is presented.

## Methods

### Aims

#### Primary aim

The primary aim of the MEMORA cohort is to study the relationship between patient characteristics and functional autonomy change over time among patients attending a MC, including neuropsychological performance, pharmaceutical and non-pharmaceutical therapy, clinical and sociodemographic characteristics.

#### Secondary aims

The secondary aims are to investigate the factors associated with cognitive performance and its change over time, and those associated with behavioral disorders.

### The MEMORA cohort design

The MEMORA cohort is a prospective, open study, started in November 2014 and conducted in a context of routine care (Table 1). The study includes patients over a period of 6 years, and each patient is followed for 3 years. The data collection is carried out throughout the care pathway. At the first visit, patients undergo a clinical examination with a medical specialist (neurologist, geriatrician, or psychiatrist). Evaluations are performed at baseline and include functional autonomy level, cognitive performance and presence and severity of behavioral disorders; depending on their cognitive status patients may be referred to a neuropsychological examination. Patients are then routinely followed-up and data are prospectively collected in an electronic Case Report Form (eCRF) using the Easily® software (University Hospital of Lyon, France); the interval between visits is 6 months to 1 year as planned in routine care by the physician in charge of the patient. The number of follow-up visits per patients has not been determined in advance and may vary from a patient to another.

**Table 1:** Flow diagram of the MEMORA-cohort

Timepoint	*Study periods*		
	*Enrollment*	*Initial assessment*	*Follow-up*
	- *t1*	0	*ti:delay to 6 months to 1 year*
Enrolment:			
Eligibility screen	X		
Information to patients/careviger	X		
Collection of non-opposition to participate	X		
Date of the visit	X	X	X
Assessments:			
*Primary outcome:*			
Functional autonomy level (IADL, DAD-6)		X	X
*Secondary outcomes:*			
Global cognitive performance (MMSE)		X	X
Behavioral disorders (NPI)		X	X
*Characteristics*:			
Sociodemographic data (age, gender, educational level, socio professional categories, marital status, geographical location)		X	
Relationship with the primary caregiver		X	
Current lifestyle		X	
Patient protection measure		X	
Neuropsychological evaluation		X	X
Diagnosis and stage		X	X
Comorbidities, lifestyle habits		X	
Pharmaceutical therapeutics		X	X
Non-pharmaceutical therapeutics		X	X
Caregiver burden (Mini-Zarit)		X	X

### Study sites and population

The MEMORA cohort includes patients attending the MC of the Charpennes Hospital, Villeurbanne, France since 2014, and has been extended since 2017 to the MC of the Dugougon Hospital, Lyon, France. The MC role is to offer evaluation and follow-up for patients with cognitive disorders, generally referred by a general practitioner or medical specialists.

Inclusion criteria are: attending a medical appointment in the MC, living at home or in retirement facility. The patients are informed of the study and its objectives and are given the opportunity to object to participation. Exclusion criteria are: hearing or visual impairment preventing cognitive assessment, institutionalization, being under legal protection.

#### Ethical and legal considerations

Information is individually provided to the patients and caregivers at inclusion. The MEMORA cohort protocol (clinicaltrial.gov number NCT02302482) has been approved by the regional ethics committee (*Comité de protection des personnes Sud Est III*) on July 29, 2014. Data processing has been approved by the national data protection commission.

### Primary outcome

The primary outcome of the MEMORA cohort is the change in the level of functional autonomy that is assessed using the 8-item version of the Lawton Instrumental Activities of Daily Living (IADL) score, and the 6-item version of the Disability Assessment for Dementia scale (DAD-6) [17, 18]. In the MEMORA study, the functional scales are collected during an interview of the primary caregiver or the patient with a physician, a nurse, or a psychologist. The change in functional autonomy will be measured using successive scores measured during patient follow-up visits at the MC.

### Secondary outcomes

The secondary outcomes include global cognitive function measured using the Mini-Mental State examination (MMSE) [19], and behavioral disorders measured using the Neuropsychiatric Inventory (NPI) [20]. The MMSE score ranges from 0 to 30 (optimal cognitive performance) and is collected during an interview with the patient by a physician, a nurse, or a psychologist. The NPI score ranges from 0 to 144 (a higher score indicating a greater number/severity of disorders) and is collected during an interview with the primary caregivers by a physician, a nurse, or a psychologist.

### Patient characteristics and evaluations

The patient characteristics and evaluations collected in the MEMORA study are listed in Table 1. Diagnosis stage and etiologies are determined by the medical specialist in charge of the patient (neurologist, geriatrician, or psychiatrist). Patients with a subjective cognitive complaint and absence of objective evidence (i.e. normal neuropsychological performance), are considered having SCD [16]. Mild and major neurocognitive disorders (NCD) are identified using the Diagnosis and Statistical Manual of mental disorders (DSM-V) nomenclature [15]. Previous medical history, family history of dementia, and comorbidities are also collected. The neuropsychological tests are chosen in a set of 142 tests allowing examining different cognitive functions (memory, executive functions, and instrumental abilities) by the psychologist, based on the patient’s cognitive status and the patient’s complaint or his entourage. The pharmacological drugs used by the patient are collected from the current general practitioner prescription and the specialist prescription at the MC. Home services, such as nurse care, day care admission, speech therapy, physical therapy, psychological support, cognitive rehabilitation or other non-pharmacological treatments are also collected. The caregiver burden is evaluated for the main caregiver who accompanies the patient at the MC using the mini-Zarit questionnaire [21].

#### Sample size

This study is designed to be descriptive rather than analytical. Based on the number of patients attending a memory consultation in the MCs and meeting the inclusion criteria, the number of patients that can be included in the study has been estimated at 1130 per year. As the means available allow to plan an inclusion for 6 years, the sample size is estimated at 6780 patients. With an expected loss of follow-up of 20% in the context of patients with cognitive disorders, the corrected sample size reaches 5650 patients. In case an association between one factor of 2 categories and the outcome change over time is to be assessed, this sample size would allow to detect an effect size of 0.1 between the 2 patients categories, at a risk alpha of 0.05, with a power of 96%. In case of factor with more than 2 categories, the power would reach 99%. This number of subjects seems sufficient to allow to assess the associations between various factors and the outcomes of the primary and secondary objectives.

### Data management and statistical analyses

Data are monitored by a clinical research associate (CRA). Inconsistencies will be reported to the study investigators in order to decide whether the data should be corrected or considered as missing data. Any changes in the data will be reported.

#### Descriptive analyses

A flow-chart will present the number of patients included at baseline, and the number of patients with follow-up visits. Characteristics of the study population and proportions of missing values will be reported. Patient characteristics will be described using mean and standard deviation or median and interquartile range for quantitative variables, and frequencies and distribution for categorical variables. Comparison of baseline characteristics between patients with complete follow-up and those with attrition will be performed. In addition, methods for handling missing data will be used such as multiple imputation, by using mixed model or auxiliary variable when appropriate [22, 23].

#### Primary analysis

Univariate and multivariate analyses will be conducted to assess the relationship between the patient characteristics and the change in functional autonomy. Linear regression and ANOVA will be performed to assess the cross-sectional relationship between patient characteristics and functional autonomy scores. The successive assessments of the functional autonomy scores, considered as the dependent variables, will be modelled in a generalized linear mixed model to assess the longitudinal relationship between patient characteristics and change in functional autonomy [24–26]. The analyses will be adjusted for potential confounding factors when appropriate. An alpha level of 0.05 will be used for statistical significance, and tests will be bilateral.

#### Secondary analyses

Similar statistical methodologies will be applied to answer the secondary objectives. The successive measures of the MMSE and the NPI scores will be considered as the dependent variables of the models.

#### Data monitoring

The successful completion of the study is ensured by the CRA. The CRA also ensures compliance with the study protocol.

#### End of protocol

Patients are excluded from study follow-up if they no longer wish to participate at any time during the conduct of the study. However, as indicated in the information letter to the patients/caregivers, the data collected before exclusion may be used as part of the study.

#### Confidentiality

The nominative patient information the enabling follow-up to be conducted is kept in a separate file th at does not contain clinical data. The access to the nominative information is protected by a password and confidentiality is guaranteed by the study.

#### Protocol amendments

Any important modifications requiring a new ethics committee approval will be communicated in future publications. The potential impact of protocol modifications on the results will be discussed as appropriate.

#### Dissemination policy

The results of the primary and secondary objectives will be published in peer-reviewed journals. All authors of future publications will have to meet the criteria for authorship stated in the Uniform Requirements for Manuscripts Submitted to Biomedical Journals by the International Committee of Medical Journal Editors.

## Discussion

The MEMORA cohort is conducted to investigate factors associated with progression of functional autonomy over time among patients with SCD or NCD in a real-life context. The data sources of the MEMORA cohort come from medical records and study-specific assessments. The real-life context of the study allows the inclusion of a large sample of patients at various stages of cognitive disorders and different management which can potentially influence the change over time of disorders related to NCD. This study can therefore been seen as a complementary approach to randomized controlled trials, which provide a higher level of evidence when well conducted but still represent high costs [27]. The results of this study should be beneficial to patients through the identification of profiles of at-risk patients and, later, targeted interventions could be assessed and proposed [28, 29]. Furthermore, the functional autonomy impairment that occurs during the course of NCD appears as a main factor associated with caregiver burden and rising societal costs, and therefore limiting its progression is likely to improve caregiver quality of life and better resource management [30–32]. The MEMORA study represents also an essential approach to assist the policymaker and healthcare decision-makers by providing results from a real-life context [33].

In terms of generalization of future results to other populations, the specificity of the study setting has be taken into account as the MCs are specialized in the ADRD diagnosis and management, providing early diagnosis and in-depth investigation. More generally, the limits associated with ADRD studies such as potential biases have been described [34], and will be taken into account when the results will be interpreted. Measurement and classification bias, referring to error in evaluation or classification of patients, could also occur and lead to reduce the reliability of the results. To limit these biases, training and information are regularly provided to the medical staff.

## Conclusion

The MEMORA-cohort conducted in a context of routine care may help to identify the factors associated with functional impairment related to progressive neurocognitive disorders, and improve the understanding of functional and cognitive impairment as well as behavioral disorders over time. This may eventually lead to propose interventions on potentially modifiable factors to improve management and delay functional dependence of ADRD patients.

## Data Availability

The final dataset of the MEMORA study will not be publicly available due to regulations and agreements obtained to perform the study, but will be available on reasonable request after publication of the primary objective. Data requests can be submitted to the researchers at the Memory Research Centre of Lyon (CMRR of Lyon, Charpennes Hospital, University Hospital of Lyon, Villeurbanne, France).

## Abbreviations

AD: Alzheimer’s disease
ADRD: Alzheimer’s disease and related disorders
CRA: Clinical research associate
IADL: Instrumental abilities of daily living
MC: Memory centers
MMSE: Mini-Mental State examination
NCD: Neurocognitive disorders
NPI: Neuropsychiatric inventory
SCD: Subjective cognitive decline
